# Recent Studies on Novocain

**Published:** 1908-10-15

**Authors:** Richard H. Riethmuller

**Affiliations:** University of Pennsylvania


### RECENT STUDIES ON NOVOCAIN.

BY RICHARD H. RIETHMULLER, PH.D., UNIVERSITY OF PENNSYLVANIA.

In August 1906, the *Dental Cosmos* (on page 882) published a note on "Novocain" which was discovered by Uhlfelder and Einhorn and put on the market by chemical works of Meister, Lucius & Bruening, at Hoechst-on-Main, Germany. Drs. Braun, of Leipzig, Brandt, Sachse, Port, Luniatschek, Dietze, Dependorf and Bieberfeld of Germany, and Messrs. Pinet and Jeay of France, immediately began a series of very successful experiments with this new anesthetic. Their good results have been corroborated by a large number of dentists, who prefer it to cocain.

In *L'Odontologie*, March 30, 1908, M. A. Thioly-Regard, of Geneva, also published a study on novacain.

Novocain—or chlorhydrate of paraminobenzoyl-diethylamino-ethanol—is an alkaloid seven times less toxic than cocain and three times less toxic than stovain, which exerts no detrimental influence on circulation or respiration. It is soluble in one part of water to one part of novocain, and the solution can be sterilized by boiling, ebullition having no effect upon its anesthetic properties nor altering its chemical composition. Dr Braun of Leipzig has used novocain for various surgical operations, injecting 0.25 centigram of a 2 percent solution of novocain without observing any symptoms of toxication. Drs. Danielson and Schmidt have obtained anesthesia by infiltration of a 1 to 2 percent solution, and have had satisfactory results even in the topical application of a 5 to 10 percent solution on the mucous and lingual membranes. In extreme cases, the dose may vary between 0.01 and 0.10 centigram of novocain. B. Sachse uses for dental extractions a solution of 2 percent, of which he injects frcm 1 to 5 cubic centimeters (17 minims to 85 minims), to which a sufficient quantity of adrenalin chlorid 1:1000 is admixed. A 1 percent solution produces sufficient anesthesia for sensitive dentin, for separating and filing the teeth, and for resection of the apex of the root.

Dr. Julius Misch, of Berlin, reports 300 cases of novocain injection combined with adrenalin chlorid for sensitive dentin and extraction of roots. The patients never felt any pain during the injection nor was there any noticeable injury to the gingival tissue. Even in cases of inflamed gums, the action of the anesthetic was sufficiently effective. Bieberfeld affirms that novocain, far from weakening the action of the adrenalin, increases its action, his statements being confirmed by the experiments of Heinecke and Lowen. Novocain anesthesia is prolonged by the admixture of adrenalin chlorid; novocain has no effect whatsoever on the vaso-constrictor properties of adrenalin chlorid. With a weak solution of novocain, the duration of anesthesia is a much longer one than with a higher dose of cocain solution. Ischemia of the gums is not as pronounced as it is with cocain. Sachse observed that the sockets after extracting bleed more profusely. Misch has corroborated that the alveolar anemia is not so noticeable with novocain-adrenalin as it is with cocain-adrenalin. These points are very important, because anesthesia combined with prolonged extensive anemia is dangerous to the life of the tissues and the organs depending upon their circulation. Cocain-adrenalin injection very much endangers the life of the peri-cemental membrane and the pulp, while with novocain-adrenalin the danger of mortification of these tissues is considerably lessened. One to 3 drops of a 1000 solution of adrenalin chlorid, added to each cubic centimeter (17 minims) of novocain is too strong. The best results will be obtained if one drop of a 1:4000 solution of adrenalin chlorid is added to each cubic centimeter of novocain. The injection of this solution will produce an anesthesia lasting from one-half to three-quarters of an hour. For numerous injections Dr. Misch has used one of the following formulae:

| | | |
|---|---|---|
| (A) | Novocain, | 0.10 ctg. |
| | Normal salt solution, | 10.00 ctg. |
| | Borated adrenalin | (1:4000), 10 drops. |
| (B) | Novocain, | 0.10 ctg. |
| | Normal salt solution, | 5.00 ctg. |
| | Borated adrenalin. | 5 drops |

With novocain the after-pain, which follows cocain anesthesia, is much less. Only in very rare cases was any after-pain felt and that was of little violence.

When preparing a solution of novocain-adrenalin, the desired quantity of pulverized novocain is dissolved in distilled water and sterilized by boiling, after which the desired quantity of adrenalin solution may be added. A small quantity of adrenalin solution should be kept in an amber-colored bottle with rubber stopper, ready for use. Novocain solutions may be kept indefinitely and be sterilized repeatedly by boiling, although stale solutions must be carefully filtered. The syringes and needles, which are sterilized in solutions containing sodium, should always be carefully cleaned with a normal salt solution, since sodium decomposes novocain. Various firms are putting novocain on the market in very practical form, as Meister Lucius & Bruening, of Hoechst, Markess-Sevogel of Basel, and Acker of Karlsruhe. The experiments of Ad. Witzel, Cieszinski, Liebl, Paul

Reynier (in *Revue Therapeutique Medico-chirurgicale,* November 1, 1907), G. Fischer of Greifswald (Saxon Dental Society Hanover, February 3, 1907), and Kirchner (*Deutsche Zahnarztliche Wochenschrift,* July, 13, 1907) sufficiently indicate the superior qualities of novocain for any sort of dental work that requires anesthesia. At one of the last meetings of the "Societe Odontologique de Geneve," M. A. Bardet introduced to the profession a new sterilizer for hypodermic syringes and needles, which has no injurious effect on the metal. This solution is as follows:

| | |
|---|---|
| Lime-water, | 1 liter. |
| Tricresol, | 20 grams. |

Lime-water is prepared by dissolving 2 grams of calcium hydrate in 1 liter of distilled water; allow to stand a day or two and shake occasionally, after which it may be filtered and the tricresol added.

The merits of novocain were also discussed in the meeting of the Netherlands Dentists' Association of June 29, 1907 (*Tijdschrift voor Tandheelkunde,* Utrecht, May 15, 1908). Dr. Boelger reported forty-three cases, in only six of which the patient felt pain, while in four cases the results were doubtful. The injections have no noticeable effect on blood circulation or respiration. According to Fischer (*Deutsche Monatsschrift fur Zahnheilkunde,* April 1, 1907), the anesthesia, with novocain is absolutely reliable. Novocain is six to seven times less toxic than cocain and three times less than stovain. In a watery solution of 1 to 1 it can be heated to 120 degrees C. without harm to its anesthetic properties; stale solutions can be sterilized again by boiling. Its anesthetic effect can be increased by the addition of suprarenin. Hamacher uses a 4 percent solution, Fisher a 1.5 to 2 percent solution with an admixture of 2 to 4 drops of a 1:5000 suprarenin solution. Dr. Boelger recommends the following solution:

| | |
|---|---|
| Novocain, | 0.750 |
| Thymol, | 0.033 |
| Sod. chlor., | 0.450 |
| Aqua destill., | 50.000 |

The thymol acts as a preservative.

To two cubic centimeters (34 minims) of this solution, three drops of a 1:1000 suprarenin solution are added before the injection.

The general interest taken in novocain by both the dental and medical profession is furthermore manifested by an article of Dr. Struthers, in the *Edinburgh Medical Journal* for February 1908. During the months preceding this publication, the author has made use of novocain clinically for inducing local anesthesia by subcutaneous injection, and as he has found that the claims made for it seem well founded, he thought it might be of interest if he indicated briefly the evidence which his experience has afforded. His remarks are based on some eighty-six cases in which he has used novocain and carefully noted the results, contrasting them with those obtained from the use of cocain and eucain in some hundreds of similar cases. These results have been uniformly good, and although the number of cases may seem small on which to base an opinion, he is inclined to believe that novocain is at least of equal and probably of greater value as a local anesthetic than cocain or cocain for subcutaneous use.

In the first place, the author states that the drug is very soluble, and that its solutions are stable and may be repeatedly sterilized by boiling without in the least losing their power of inducing anesthesia. He has tested this by making up a large quantity of a stock solution and using it over a period of several months, sterilizing it over and over again during that time. The solutions combine well with solutions of adrenalin, and do not in the least interfere with the vasoconstrictor action of the latter.

For infiltration anesthesia the author has found that a solution of novocain in 0.75 percent saline solution to the strength of 1:400, plus one drop of the ordinary 1:1000 adrenalin solution to every 2 or 3 drams of solution used, the strength of solution originally recommended by Braun, answers admirably. It corresponds to what may be termed the standard for infiltration of 1:1000 cocain, but has the advantage that it may be used in larger quantities; for while the limit of safety is reached when about four ounces of the cocain solution have been used, at least six ounces and probably more of the novocain solution may be employed for an adult without any risk.

In addition to this, it diffuses readily and acts as quickly as the cocain solution, anesthesia being satisfactory in ten to fifteen minutes after the injection is complete, and for this reason novocain is to be preferred to eucain, for the latter may require as much as half an hour to take full effect. The duration of the anesthesia is always more than an hour, often as long as three or four hours. After it has passed off there is often, as with other drugs, a variable amount of burning and smarting pain in the wound and the author has seen no reason to infer that this is either greater or less than with cocain, eucain, etc. Sloughing of the skin, which occasionally follows the use of local anesthetics, particularly eucain and stovain, the writer has never seen, nor has he heard it reported after the use of novocain.

Several of the author's operations were done in children as young as five and six years of age. In no case was there any sign of toxic symptoms arising from the use of the novocain-adrenalin solution. Novocain has proved as satisfactory for regional as for infiltration anesthesia, and in conclusion the author states that he believes the advent of novocain a real, though perhaps slight advance in the possibilities attending the use of local anesthesia by subcutaneous injection. It is stable, readily sterilized, unirritating, and efficient as a local anesthetic when combined with adrenalin, and can apparently be used in doses to meet all requirements without any fear of serious toxic symptoms arising.

In an article on "Local Anesthesia by Novocain," published in the *British Medical Journal* of May 18, 1907, Dr. J. W. Pare reports seventy cases, in all of which the application of novocain was most satisfactory. His procedure in administering this anesthetic may prove interesting:

(1) I give the patient half a pint of warm carbolic lotion with which vigorously wash out the mouth, so as to reduce its septicity.

(2) The all-metal hypodermic syringe (with vulcanized "vela" rubber packing), needle, glass measure, glass mortar, glass mixer, are all placed in hot water to raise the temperature to that of the patient.

(3) If the patient be neurotic, a swab containing a 5 percent. solution of novocain is put on the gum for five minutes, so as to render the latter insensitive to the insertion of the needle.

(4) One or two novocain tablets, each containing one-third grain of novocain, are dissolved in 33 minims of hot water to make either a 1 or 2 percent. solution respectively.

(5) Hot water is passed through the syringe and needle, the novocain solution is drawn up into the syringe, and the syringe inverted to allow the air bubbles to rise into the needle from which they are easily displaced by pressing the piston.

(6) If an upper tooth, for example, is to be extracted, the needle is inserted into the gum one-eighth inch above the cervical margin, and a drop or two of novocain solution is forced in; I keep the needle in position a few seconds to allow the anesthetic to act before forcing any more fluid into the tissue. After forcing in the solution, the needle should not be immediately removed, because if this is done then, owing to the pressure being greater in the gum than outside, some of the fluid will escape. If the position selected be a good one (between the roots is better than over them) the gum will gradually blanch and become of the consistence of cheese; the needle should then be removed and again inserted just inside the periphery of the blanching, and so on, till the whole of the gum on both sides of the tooth is anesthetized. I do not hurry the injection, but take about three to five minutes for the process. The tooth can then be extracted. I have so far never used more than three tablets (one grain of novocain) at one sitting.

In concluding, Dr. Pare summarizes the advantages of novocain as follows:

(1) It produces a perfect local anesthesia.

(2) The duration of the anesthesia is longer than that of cocain.

(3) Even in strong solutions it does not irritate the tissues.

(4) It is at least equal to cocain in anesthetic power.

(5) It is many times less toxic than cocain, in comparison with which it can be used in larger doses with perfect safety.

(6) It is very constant in its action.

(7) It does not produce shock, cardiac or respiratory failure, after-pain, nor sloughing of the gums.

(8) It can be given immediately after food.

(9) It is not a secret preparation, but a substance of known and definite chemical composition.

(10) It is cheaper than most proprietary anesthetics preparations.

A very interesting case, which goes to show that the experiments with novocain are by no means finished, is cited in the *Deutsche Zahnartzliche Wochenschrift*, June 27, 1908. Dr. Guido Fisher of Greifswald reports a case of narcotic slumber after local anesthesia with novocain, which had been administered to a strong and healthy woman of thirty-six, for the purpose of extracting the gangrenous roots of a lower second molar. Anesthesia was obtained by an injection of three cubic centimeters (50 nimims) of a 2 percent. novocain-thymol solution, to which three drops of a synthetic suprarenin solution (1:1000) had been added immediately before injecting. The injection, as is usual in patients with a good constitution, caused no pain to speak of. On account of the difficult diffusion in the mandible, it was estimated that the anesthetic would require fifteen minutes to take full effect; in the meantime two cavities in teeth in the left maxilla were excavated.

About one minute affer the injection, the patient felt an extensive numbness in the entire half of the left mandible, and after five minutes was unable to feel the rinsing glass at the left side of her lips. A the same time the pulse was accelerated for about one or two minutes. Then the patient fell into a sort of doze and seemed to have difficulty in keeping her mouth open for the preparation of the defective upper teeth. Pulse and respiration were soon normal again, and the patient gave the impression of a comfortably slumbering person. As if in an hypnotic dream, she followed every one of the operator's instructions, rinsed, opened and closed her mouth, without opening her eyes or being conscious of what she was doing. The upper cavities were filled with amalgam, excavation producing no pain whatever, although the bur came very near the pulp. Twenty minutes after the injection two badly decayed roots were removed. Immediately after the extraction, the patient raised herself as if startled, opened her eyes and thoroughly rinsed her mouth as directed. She appeared to be completely changed, acted normally, and stated that pain, apparently caused by pressure, had startled her. The numbness in the left side of the jaw was still present, and the woman blushed when she heard that she had been asleep. She was proud of always having had an exceedingly strong constitution, and mentioned as a sort of excuse for her sleeping that her system had always quickly and intensively reacted to any medicine. She attributed her slumbering to this peculiarity, the normal dose evidently having affected her much more strongly than it would an average person. She did not know what had happened while she was asleep and was glad to hear that her two upper premolars had been filled. The patient left the surgery fully conscious and feeling very well.

This case of short "hypnotic" slumber was evidently due entirely to the effect of the novocain. Erotic affections such as have been frequently observed during general anesthesia and occasionally during local anesthesia with chlorid of ethyl and cocain seemed to be entirely wanting in this case. Nevertheless light narcotic sleep after application of novocain might occur in a sexually easily excitable person; again a note of warning must be sounded to always have a third person present, not only in general but also in local anesthesia, in order to avoid all possible danger of suspicion.

The extraordinary effect of novocain in this case must be considered as a mild intoxication, or irritation rather, of the central nervous system, brought about by the small dose of 0.06 novocain. It is remarkable that not one of the toxic phenomena of novocain heretofore observed and tested by F. Liebl (*cf. Munchner Medizinische Wochenschrift*, 1906, No. 5), was present in this case. Four minutes after injecting 0.75 gram of a warm 10 percent. solution into his right thigh, Liebl felt "a sudden peculiar warmth in his entire body, especially in the region of the liver, slight indisposition and nausea, and general restlessness. There was no noticeable change in pulse nor complexion. Two minutes later slight deafness in the left ear was perceived. Optic disturbances: Accommodation possible only with greatest effort, especially in the left eye; double images. Thirteen minutes after the injection, light piercing headache in the left occiput. Seven minutes later, paresthesia in the region of the left scaphoid bone." After an indisposition of half an hour, normal comfort gradually returned.

The slight acceleration of the pulse shortly after the injection in the present case is probably to be attributed to the suprarenin rather than to the anesthetic, since it has never before been observed with solutions of novocain thymol. Liebl also remarks explicitly that pulse and complexion showed no change.

It is interesting and important that novocain which has an extremely light toxic effect upon the tissues and can be borne even pure without any disturbances, may occasionally call forth an irritation of the central nervous organs even if applied in a dose far below the maximal. A system which, as in the case presented, apparently reacts to the most minute chemical irritations and whose protoplasm is exceedingly sensitive, may individually require a maximal dose far below that heretofore generally accepted, although the quantity of solution was larger. For, whether intoxication of the central nervous system with novocain is manifested, and in what intensity, depends, according to H. Braun, not only upon the dose of novocain injected, but also upon the time in which it was injected. If the solution gets into the blood suddenly, that is, in concentrated form, then that dose can have immediate toxic effects, while, if injected slowly, that is, in dilute form, or if administered at intervals, the novocain solution would produce not even the slightest indications of intoxication of the central nervous system, because the concentration of novocain in the capillaries of the nervous system never exceeds the toxic degree of concentration.

Dr. Klein, also, reports having observed five cases of pronounced toxic effects after novocain (*Deutsche Zhanarztliche Wochenschrift*, page 138, 1908). Two of these cases were due to a complication of functional disturbances in the heart, lack of power of resistance of the entire system and abnormal menstruation; in the other two cases, however, novocain alone was to be considered responsible for phenomena of collapse. Dr. Klein's two cases as well as the case presented by Dr. Fisher must be regarded as exceptions to the rule. After three years' experience with novocain, Dr. Fischer is inclined to once more emphazise its advantages over cocain, although in very rare cases it has manifested unpleasant secondary phenomena. Yet these secondary phenomena have been of such a mild and tolerable character that the good reputation of novocain in regard to its toxicity has not been impaired. For it is a local anesthetic which only when applied in a dose of 0.75 gram, a quantity which is out of the question in local anesthesia, and only under conditions especially favorable for resorptive effects (10 percent. solution at body warmth), showed phenomena of slight yet relatively harmless intoxication, which bears absolutely no comparison with the uncanny phenomena of cocain poisoning. Even 0.4 gram of a 10 percent. solution produced no toxic effects. Toxic cases with novocain have been so rare and of such a singular nature that its use on the whole may be called most satisfactory. Among 5000 cases of novocain injection, Dr. Fisher cannot cite one single case of serious intoxication, while the grave accidents with cocain are still fresh in our memory. Nevertheless the few interesting abnormal conditions after the use of novocain only prove the old rule that the patient's individuality plays an important role in every remedy.

Cocain not only often causes general disturbances, but it not unfrequently—especially if a not quite pure solution has been applied—starts a process of local gangrene in the region of the tissues anesthetized. This has never been observed after novocain; also after-pain has been reported only exceedingly rarely after novocain injections. The formation of edema, especially after the extraction of badly decayed teeth, can no more be prevented by novocain than by any other anesthetic.

For his private use Dr. Fischer has now from time to time prepared a quantity of solution after a formula which only slightly varies from the one recommended by him two years ago; in its essence it coincides with that of Dr. Boelger:

| | |
|---|---|
| Novocain, | 1.000 |
| Thymol, | 0.033 |
| Sod. chlor., | 0.450 |
| Aqua destill., | 50.000 |

This solution is preserved in dark bottles, closed with rubber stoppers and holding 850 minims. The solution, which is prepared at boiling heat, if kept in well-closed bottles, remains for months as clear as it was on the first day, and, when needed, is poured into a small glass dish, in which one drop of fresh 1:1000 synthetic suprarenin solution is added immediately before the injection. The prepared mixture must be used up immediately.

Pure novocain powder, moreover, renders excellent service in the treatment of all forms of acute inflammation of the oral cavity, especially in the therapy of painful wounds, difficult dentition, etc. In regard to surprisingly quick and lasting removal of pain and to the production of sound granulation its invariable effects are unequaled.

In spite of these in the main excellent experiences with novocain, which have been endorsed by numerous authors, this anesthetic has not been adopted by dental practitioners as generally as its merits would warrant. Unfortunately a great many well-advertised secret praparations are still on the market. It is very much to be regretted that many dentists, instead of profiting by the scientific experiments so conscientiously conducted at different dental schools, still put faith in the expensive and greatly overrated secret preparations. Novocain has proved itself much less dangerous and surely no less powerful than cocain or any secret anesthetic, and it cannot be emphasized strongly enough that local anesthesia in the majority of cases is the only indicated method for the prevention of pain. Its application, to be sure, requires a special techincal ability which seems to be lacking with some practitioners. Many dentists for the sake of convenience never fail to apply anesthesia even in most insignificant cases, and thereby forget what great risks their indifference entails for the life of their patients.

If one observes the successful efforts of general surgery in limiting general anesthesia by the introduction of lumbar anesthesia, and therewith compares the wide disagreement prevalent in the dental profession as to anesthesia, one can hardly dispel the feeling of solicitude for the further development and final perfection of this most important branch of dental science.—August 1908, *Cosmos*.

